# A System of Legal Medicine

**Published:** 1895-02

**Authors:** 


A System of Legal Medicine. By Allan McLane Hamilton, M. D., consulting physician to the insane asylums of New York City, etc., etc., and Lawrence Godkin, Esq., of the New York Bar, with the collaboration of Prof. James F. Babcock, Lewis Balch, M. D., Judge S. E. Baldwin, Louis E. Binsse, Esq., C. F. Bishop, Esq., A. T. Bristow, M. D., B. F. Cardozo, Esq., C. D. Chaddock, M. D., A. F. Currier, M. D., C. L. Dana, M. D., George Ryerson Fowler, M. D., W. T. Gibb, M. D., W. S. Haines, M. D., F. A. Harris, M. D., W. B. Hornblower, Esq., Charles Jewett, M. D., P. C. Knapp, M. D., R. C. McMurtrie, Esq., C. K. Mills, M. D., J. E. Parsons, Esq., C. E. Pellew, E. M., Judge C. E. Pratt, W. A. Purrington, Esq., B. Sachs, M. D., F. R. Sturgis, M. D., Brandreth Symonds, M. D., V. C. Vaughan, M. D. Complete in two royal octavo volumes of 700 pages each, illustrated. New York: E. B. Treat, 5 Cooper Union, 1895. In substantial cloth binding, per volume, $5.50. In full sheep, uniform law style, per volume, $6.50. Sold by subscription. Orders taken only for the complete work.

A notice of the first volume of this superb work appeared in The Homœopathic Physician for July, 1894, page 219. The second volume is now before us. The list of contributors to this great work includes the names of thirty of the most distinguished writers and authorities upon medical jurisprudence in America, with upwards of five thousand citations and cases. As a book of reference it will be found an invaluable help to medical men and to those of the legal profession who desire the aid of the most advanced and sound opinions of practical students of forensic medicine. So much opprobrium has been attached to the word "expert" that the spirit which so often impels men to go into court and become ardent partisans finds no place in this system, and it has been the aim of the editor and his colleagues to give the work a decided judicial and impartial tone, so that it may be consulted with confidence by all as an authority of the first order.

Until recently the contributions in the United States to the literature of medical jurisprudence have been exceedingly meagre, if we may except Beck's classical but antiquated treatise, and other works limited in scope. From necessity, it has been the custom to consult foreign books, which were written for the benefit of transatlantic readers, and are in many respects inapplicable to our methods, and not in conformity with the legal usages of this country. Therefore, the appearance of an American treatise of this character will be especially timely and welcome.

The editor has aimed to make the work under consideration a repository of the most advanced ideas and valuable cases, and, except when the latter are unique, indispensable, or especially pertinent, it has been his aim, and that of his associates, to avoid threadbare material, and to illustrate the articles by new examples. The scope of the work is necessarily very great, but it is trusted that its contents will be found to be practical and concise. Extraneous matter is dispensed with, and the reader is spared dry and uninteresting details and a repetition of valueless decisions. A feature of Hamilton's *System of Legal Medicine* is the presentation of a large amount of new experimental research by contributors who have actually figured repeatedly in notable cases in civil, criminal, and probate courts in various parts of the country.

